# Quantitative causal selection patterns in token causation

**DOI:** 10.1371/journal.pone.0219704

**Published:** 2019-08-01

**Authors:** Adam Morris, Jonathan Phillips, Tobias Gerstenberg, Fiery Cushman

**Affiliations:** 1 Psychology Department, Harvard University, Cambridge, MA, United States of America; 2 Psychology Department, Stanford University, Stanford, CA, United States of America; Middlesex University, UNITED KINGDOM

## Abstract

When many events contributed to an outcome, people consistently judge some more causal than others, based in part on the prior probabilities of those events. For instance, when a tree bursts into flames, people judge the lightning strike more of a cause than the presence of oxygen in the air—in part because oxygen is so common, and lightning strikes are so rare. These effects, which play a major role in several prominent theories of token causation, have largely been studied through qualitative manipulations of the prior probabilities. Yet, there is good reason to think that people’s causal judgments are on a continuum—and relatively little is known about how these judgments vary quantitatively as the prior probabilities change. In this paper, we measure people’s causal judgment across parametric manipulations of the prior probabilities of antecedent events. Our experiments replicate previous qualitative findings, and also reveal several novel patterns that are not well-described by existing theories.

## Introduction

When something happens, people commonly ask: What caused that? Making causal judgments is often deceptively easy. We naturally conclude that the lightning strike caused the forest fire; the last-minute goal caused the sports team’s victory; or the scandal caused the political candidate’s defeat. These kinds of causal judgments are also very important, structuring how we understand and interact with our environments.

Yet, there are certain things that people are reluctant to label causes of an event, even though the event clearly depended on them. For instance, it is less natural to conclude that the forest fire was caused by the presence of oxygen, or by the lack of rain, or by the arsonist’s birth, even though the all of these events were necessary for the fire to happen.

This phenomenon is widespread: When an outcome occurs, people consistently judge certain seemingly-relevant events more causal than others [1–4]. This phenomenon is sometimes labeled “specific” causation [5, 6], or “causal selection” [7–9]. Understanding how and why people do this has been a central focus of philosophical and psychological inquiry for decades. (In this paper, we focus exclusively on token causation, where people judge the causes of a singular outcome, rather than type causation, where people judge the causes of an outcome in general).

When attempting to understand why some causes stand out while others recede, a key fact is that this distinction is not dichotomous, but rather *graded* [10]. Rather than judge one event entirely a cause and the others entirely not a cause, people appear to judge causation on some kind of continuum. For instance, people might say that the arsonist was most causal; the lack of rain somewhat causal; and the arsonist’s birth the least causal. Although gradation was commonly assumed in many models of type causation [11], the graded nature of token causation has only more recently attracted attention [3, 4, 12–14].

Despite its importance, however, our empirical understanding of gradation in token causal judgment is limited. Many studies have investigated how qualitative shifts in the parameters of causal systems (e.g. the prior probability of the antecedent events) produce qualitative shifts in causal judgment (see [3] for an overview), but fewer have mapped the *quantitative* relationship between those parameters and the resulting judgments. (For a notable exception, see [15–17]). Here, we contribute to closing this empirical gap. We present people with two basic causal systems, systematically manipulate the prior probabilities of the antecedent events in those systems, and elicit people’s causal judgments. We find that, even in the most basic systems, the quantitative form of people’s judgments can be complex, and is incompletely described by existing theories. This result highlights the need for future research on the quantitative form of token causal judgments.

### Qualitative manipulations of token causation

Several qualitative manipulations are known to affect which events people consider causal. We do not attempt an exhaustive review; rather, we follow Icard et al. [3] and highlight certain robust ways in which a key feature of a deterministic causal system—the prior probabilities of the candidate causes—affects people’s judgment.

#### Conjunctive systems

We first consider conjunctive causal systems, where multiple antecedent events were counterfactually necessary for an outcome—e.g. there was a fire which required both oxygen and a lit match. In these systems, classic normative analyses of causation would, roughly, label the necessary events as equally causal [18]. Yet, people consistently judge an antecedent event more causal if it is *rarer*—i.e. if, *a priori*, it was less likely to occur.

This effect has been demonstrated in two ways. First, when asked to select which of several necessary events was the cause of an outcome within some naturally occurring series of events, people tend to pick the rarer event. For instance, people say the match, not the oxygen, caused the fire. They do so in part because oxygen is always present, and a lit match is rarer [1]. This basic finding has been replicated in many scenarios [7, 19, 20].

Second, researchers have directly manipulated the prior probability of antecedent events, and found that making an event rarer causes people to assign it more causality. For instance, Icard et al. gave people vignettes like the following [3]:

Professor Smith works at a large university. At this university, in order to get new computers from the university, faculty like Prof. Smith must send an application to two administrative committees, the IT committee and the department budget committee. Prof. Smith will be able to get her new computers if the IT committee AND the department budget committee approve her application.

Icard et al. manipulated the prior probability of one of the causes by telling participants that the budget committee either “almost always” or “almost never” approves applications. Then, participants were told that both of Prof. Smith’s applications were approved, and she received a computer. Participants were asked the extent to which they agreed that the focal event—the budget committee’s approval—caused Prof. Smith to receive the computer. Icard et al. found that people agreed substantially more when the budget committee almost never approves applications. This result, along with many similar results, demonstrates that rarer events are often considered more causal. Following others, we refer to this effect as “abnormal inflation”.

A less thoroughly explored effect is that, in conjunctive systems, people also judge an antecedent event *less* causal if the *other* necessary events are rare [21]. For instance, if the budget committee almost never approves applications, then people judge the IT committee less causal; the budget committee “supersedes” the other potential causes. This is known as the supersession effect [3, 21].

#### Disjunctive systems

In disjunctive causal systems, where multiple events are each sufficient to produce an outcome, these effects change. Here, people appear to judge events more causal if they are *less* rare—an effect labeled “abnormal deflation” [3]. For instance, if either committee can unilaterally approve Prof. Smith’s application, then the stingier committee is judged less causal. Moreover, the supersession effect appears to be eliminated in disjunctive structures [3, 21]. These findings, however, are less well-studied.

#### Summary

In sum, we’ve highlighted four causal selection effects in token causal judgment: abnormal inflation and supersession in conjunctive systems, and abnormal deflation and no supersession in disjunctive systems [3]. There are two features of these results worth noting. First, these causal selection effects occur even when people already know the underlying causal structure. Many models capture how people infer unobserved causal structures from sparse data [11, 13, 14, 22], but these models are not designed to explain why people consider some variables more causal than others in the situations we study here. Our focus is on causal selection effects when people already know the causal structure.

Second, these causal selection effects have typically been studied with qualitative manipulations of the rarity of the antecedent events. Relatively little is known about how token causal judgments vary quantitatively with the probability of antecedent events. This is our point of departure.

### Our experimental design

**Fig 1:**
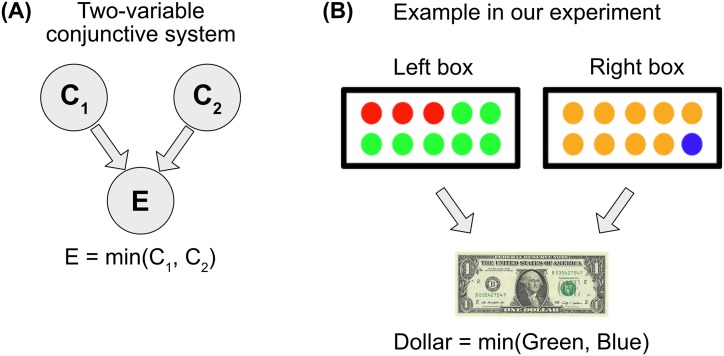
(A) Causal structure with two variables that are individually necessary and jointly sufficient to produce an outcome. The outcome equals 1 if and only if both *C*_1_ and *C*_2_ equal 1. (B) The example system used in our experiment. Joe wins a dollar if and only if he draws a green ball from the left box and a blue ball from the right box.

To examine the quantitative nature of token causal judgment, we consider a deterministic causal system in which two binary variables combine, either conjunctively (Experiment 1) or disjunctively (Experiment 2), to produce a binary outcome (Fig 1A). In two experiments, we systematically manipulated the prior probabilities of the antecedent variables taking on value 1, and asked people the extent to which they believed one of the two variables was the cause of the outcome.

As discussed above, previous research has established that the prior probabilities of the antecedent variables matter for causal judgment. However, prior work has largely restricted itself to qualitative manipulations—e.g. comparing cases where an antecedent event is rarely present to cases where it is almost always present. Here, we map the prior probability of each antecedent variable (*C*_1_ and *C*_2_) to ten values: 0.1, 0.2, …, 1. By independently manipulating *Prob*(*C*_1_ = 1) and *Prob*(*C*_2_ = 1) across the ten values, we produce one hundred different parameterizations of the causal system. We present these hundred parameterizations to people, and ask them to what extent they agree with the statement that *C*_1_ caused the outcome.

To preview our results: We largely replicate the first three qualitative findings described above (abnormal inflation and supersession in conjunctive structures, abnormal deflation in disjunctive structures), but we find a “reverse” form of supersession in disjunctive structures. Moreover, our experiments reveal nonlinear effects which, to our knowledge, are not well captured by existing theories.

The data and analysis code for both experiments can be found at https://github.com/adammmorris/causality.

## Experiment 1: Conjunctive scenario

In Experiment 1, we presented participants with the following vignette and an associated image (Fig 1B):

A person, Joe, is playing a casino game where he reaches his hand into two boxes and blindly draws a ball from each box. He wins a dollar if and only if he gets a green ball from the left box and a blue ball from the right box.

Joe closes his eyes, reaches in, and chooses a green ball from the first box and a blue ball from the second box. So Joe wins a dollar.

**Please tell us much you agree or disagree with this statement:** Joe’s first choice (where he chose a green ball from the first box) caused him to win the dollar.

The images presented the two boxes, illustrating the percentage of green balls in the left box and blue balls in the right box. By manipulating these images, we manipulated the prior probability that Joe draws a green ball or a blue ball. Since we ask people to rate the causality of drawing the green ball, we will refer to green as the “focal” variable (labeled focal) and blue as the “alternate” variable (labeled alternate). We divide the continuous space of probability into ten values: *Prob*(focal = 1) = 0.1, *Prob*(focal = 1) = 0.2, …, *Prob*(focal = 1) = 1. We divided the probability of alternate similarly. (We excluded zero because the outcome that participants actually observe is that Joe wins, which cannot happen if drawing either ball has probability zero).

For each observation of the system, we randomly selected a prior probability for focal and alternate (the two are independent), and showed the participant the corresponding image. We then told the participant that Joe wins the dollar, and asked them to rate the extent to which they agree that Joe drawing the green ball caused him to win the dollar. Participants responded on a scale from 1 (“totally disagree”) to 9 (“totally agree”). (Like others [3], we use an agreement scale, rather than a more direct “how much of a cause is the green ball?” scale, for two reasons. First, in English the phrase “how much of a cause…” is non-standard. Second, not all theories agree that causal judgments are graded. The phrase “how much of a cause” might suggest to participants that we expect them to answer in a graded way; asking the extent to which they agree with a causal statement gives them more room to freely answer in either a graded or categorical way).

### Methods

All participants were recruited through Amazon Mechanical Turk. They gave informed consent, and the study was approved by Harvard’s Committee on the Use of Human Subjects. The experiment took about 3 minutes to complete, and participants were paid $0.35 each.

We employ a within-between subjects design. Each participant was given the vignette five times, each time with a randomly chosen probability setting. (We ensured that a participant did not see the same probability setting twice). We ran *N* = 999 subjects, and collected a total of 4964 ratings. (No subjects were excluded, but some subjects did not complete all five ratings). There are 100 different probability settings (10 for focal crossed with 10 for alternate), which gives an average of about 50 individual ratings per probability setting.

### Theoretical predictions

In addition to the qualitative predictions described above (abnormal inflation and supersession in the conjunctive structure), several contemporary theories of token causation make more precise quantitative predictions in our task. In our treatment of these models, we sometimes make additional assumptions to adapt them to the present task. Our intent is not to critically evaluate the models in their exact original setting, but rather to examine the extent to which plausible instantiations of them can capture quantitative variation in the situations we study. Also, we focus primarily on the *shape* of the predicted judgment patterns (e.g. people will judge a variable more causal when it becomes rarer), rather than the absolute value of the predictions (e.g. people will judge a variable 30% causal under certain conditions). The former is more essential to each model, while the latter is influenced by many extraneous factors (how people interpret the scale, the specific parameter choices for each model, and so on), which are difficult to predict.

#### Halpern & Hitchcock

One influential theory comes from Halpern & Hitchcock [4]. Halpern & Hitchcock argue that when people evaluate counterfactuals by considering alternative possible worlds, they prefer to imagine worlds that are more normal, where unlikely things haven’t occurred [19, 20]. For instance, people tend to imagine a world in which an arsonist didn’t light a match rather than a world in which there were no oxygen in the air. Or, in our case, if it’s rare to draw a green ball but common to draw a blue ball, they would tend to imagine an alternative world in which a green ball was not drawn.

Halpern & Hitchcock argue that this observation can explain the dependence of causal judgment on the prior probabilities of the variables. To determine whether a variable *C* was counterfactually necessary for an outcome, we consider an alternate world—a “witness” world—in which the variable is absent (and perhaps some other variables have taken on different values; for details, see [4, 23]). Call this world *w*_*C*_. Halpern & Hitchcock propose that people will only judge a variable a cause of an outcome if (a) there exists a witness world in which *C* was necessary for the outcome, and (b) if that witness world—*w*_*C*_—was more normal than the actual world. Moreover, if two variables *C*_1_, *C*_2_ meet these criteria, then *C*_1_ will be considered more of a cause if $w_{C_{1}}$ is more normal than $w_{C_{2}}$.

**Fig 2:**
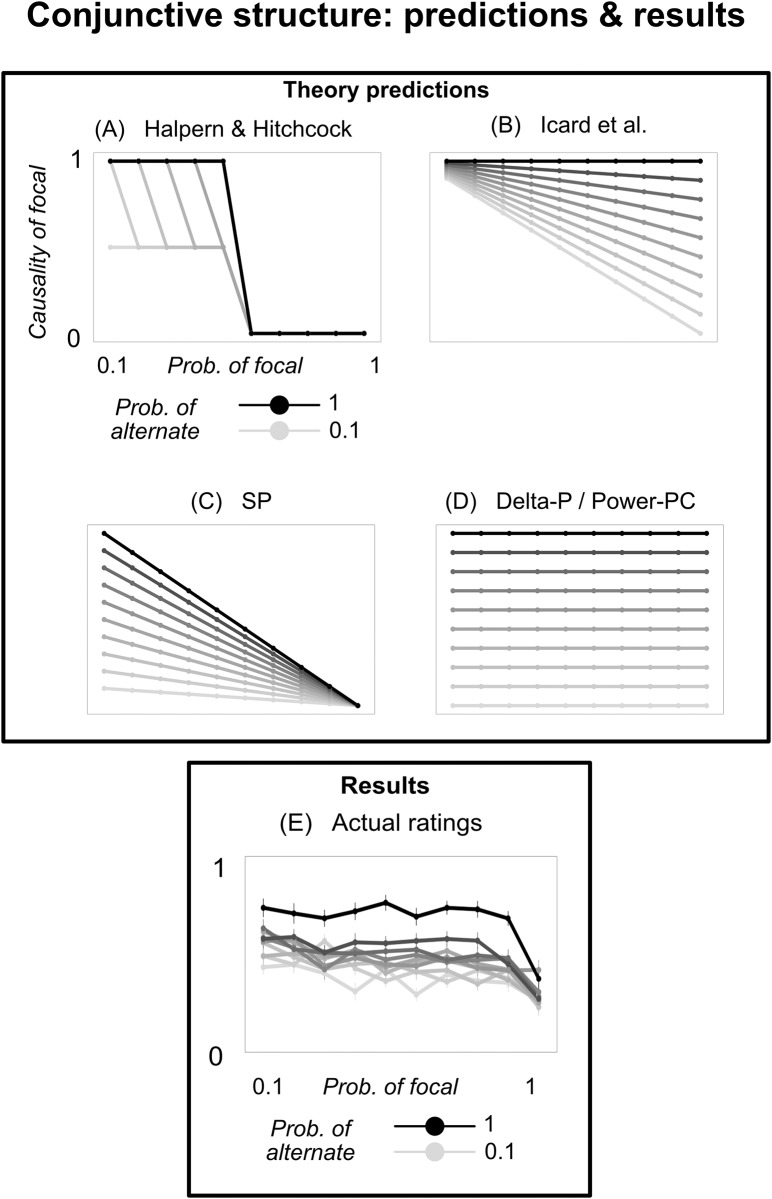
Predictions and results from Experiment 1. (A-D) Predictions from the five models we analyze. The x-axis indicates the probability of drawing a green ball; line color indicates the probability of drawing a blue ball; and the y-axis indicates focal’s predicted causal rating. (E) Empirical results; y-axis indicates people’s agreement that drawing a green ball caused the outcome. Error bars depict +/−1 SEM.

Halpern & Hitchcock’s model is not committed to a particular normality ordering over possible witness worlds (for a detailed discussion, see [24]). In our task, however, it is likely that people’s normality ordering would be based on the probability of drawing the two balls; for instance, if the probability of drawing green is greater than 0.5, then a world in which it is drawn is more normal than a world in which it is not drawn. By making this assumption, we can derive a predicted response profile in our experiment (Fig 2A). If the probability of drawing a green ball is greater than 0.5, then the green ball should never be considered a cause, because *w*_focal_–the world in which green is absent, which is the sole witness world for green (the focal variable)–is less likely than the actual world. (In the actual world, a green ball was drawn; in *w*_focal_, a green ball was not drawn. If *Prob*(focal = 1) > .5, then, all else being equal, *w*_focal_ is less likely than the actual world). This result is depicted in the bottom part of Fig 2A. If the probability of drawing green is less than 0.5 and also less than the probability of drawing blue, then green is assigned a high causal rating, because *w*_focal_ is more likely than *w*_alternate_. This is depicted in the top part of Fig 2A. Finally, if the probability of drawing green is less than 0.5 but greater than the probability of drawing blue, then green is causal but less causal than blue; it is assigned an intermediate rating.

Formally, then, the degree to which focal = 1 was a token cause of Joe’s win is:

$$\begin{array}{c} \begin{array}{c} TC_{HH}\left(\text{focal}\to \text{dollar}\right)=\{\begin{array}{c} 0\;if\;Prob(\text{focal})>.5\\ x_{high}\;if\;Prob\left(\text{focal}\right)<Prob\left(\text{alternate}\right)\leq .5\\ x_{mid}\;if\;Prob\left(\text{alternate}\right)<Prob\left(\text{focal}\right)\leq .5 \end{array} \end{array} \end{array}$$

where 0 < *x*_*mid*_ < *x*_*high*_ ≤ 1.

Halpern & Hitchcock’s model is ordinal [4], and so it cannot assign numbers for *x*_*high*_ and *x*_*mid*_. Here, for simplicity, we assume that *x*_*high*_ = 1 and $x_{mid}=\frac{1}{2}$. Our intention is not to evaluate the original model that the authors proposed. Rather, we derive a plausible implementation of their model that makes quantitative predictions in our task, and see if this derivative account can capture the overall shape of people’s judgment.

#### Icard et al.

Another theory we consider comes from Icard, Kominsky, & Knobe [3]. Icard et al. offer a variation of the counterfactual approach. They propose that, when evaluating causality, people sample a counterfactual world with probability proportional to how likely that world is. For instance, to evaluate whether focal = 1 was the cause of Joe’s win, they sample a world in which focal = 0 (he didn’t draw a green ball) in proportion to *Prob*(¬focal), and a world in which focal = 1 (he did draw a green ball) in proportion to *Prob*(focal). Then, crucially, people evaluate a different counterfactual depending on what they sampled. If they sampled a world in which focal = 0, they evaluate whether focal was necessary (holding all else about the actual world fixed); if they sampled a world in which focal = 1, they evaluate whether focal is, in general, sufficient (allowing other variables to vary).

According to Icard et al., while the probability of sampling a counterfactual world with focal = 0 is monotonically related to *Prob*(focal = 0), the two are not necessarily equal; there may be other factors that influence which worlds come to mind. This feature makes it difficult to derive precise quantitative predictions in our experiment. Nonetheless, if we make the simplifying assumption that the probability of sampling counterfactual worlds comes directly from the prior probability of green, then we can derive a predicted response profile:

$$\begin{array}{l} TC_{Icard}(\text{focal}\to \text{dollar})=\{\begin{array}{l} Prob(\text{focal}\;\text{was}\;\text{necessary})\;\text{if}\;\text{sampled}\;\text{world}\;\text{has}\;\neg \text{focal}\\ Prob(\text{focal}\;\text{would}\;\text{be}\;\text{sufficient})\;\text{if}\;\text{sampled}\;\text{world}\;\text{has}\;\text{focal} \end{array}\\ \;=Prob(\neg \;\text{focal})\cdot Prob(\text{focal}\;\text{was}\;\text{necessary})+\\ \;Prob(\text{focal})\cdot Prob(\text{focal}\;\text{would}\;\text{be}\;\text{sufficient}) \end{array}$$

In the structure of Experiment 1, focal was certainly necessary (holding all else about the actual world fixed), and, allowing other variables to vary, would be sufficient if alternative were present. Hence, this equation reduces to:

$$\begin{array}{l} TC_{Icard}\left(\text{focal}\to \;\text{dollar}\right)=Prob\left(\neg \text{focal}\right)\cdot 1+Prob\left(\text{focal}\right)\cdot Prob\left(\text{alternate}\right)\\ \;=1-Prob\left(\text{focal}\right)+Prob\left(\text{focal}\right)\cdot Prob\left(\text{alternate}\right)\\ \;=1-Prob\left(\text{focal}\right)\cdot \left(1-Prob\left(\text{alternate}\right)\right)\\ \;=1-Prob\left(\text{focal}\right)\cdot Prob\left(\neg \text{alternate}\right) \end{array}$$

The predictions of this simplified version of Icard et al.’s model are shown in Fig 2B.

#### Classic causal strength measures

Finally, we consider three classic “causal strength” measures, some of which were designed primarily to capture other flavors of causal judgments (e.g. type causation). Nonetheless, these measures make plausible quantitative predictions in the causal systems we test, and so we include them in our analysis.

The first, denoted SP, assigns causality to a variable to the extent to which observing the variable raised the probability of the outcome: *TC*_*SP*_(*C* → *E*, *u*_*i*_) = *Prob*(*E* ∣ *C*) − *Prob*(*E*) [25]. For instance, the boy throwing the ball at the window significantly raised the probability of the window shattering (compared to the prior unconditional probability of the window shattering); hence, on this view, the boy’s actions were causal. In our simple setup, this becomes: *TC*_*SP*_(focal → dollar) = *Prob*(alternate) ⋅ *Prob*(¬focal) (Fig 2C).

The second is similar, but requires causes to raise the probabilities of their outcomes, relative to a state where the cause was absent. This model, denoted Delta-P, takes the form: *TC*_*Delta*−*P*_(*C* → *E*, *u*_*i*_) = *Prob*(*E* ∣ *C*) − *Prob*(*E* ∣ ¬*C*) [26, 27]. On this causal strength measure, the boy’s throw must raise the probability of the window shattering, compared to the case where he didn’t throw the ball (rather than the case, used in SP, where it is unknown whether he threw the ball). This alternate conceptualization allows the boy to be causal even if, for instance, he throws the ball at the window every day. In our experiment, this measure becomes: *TC*_*Delta*−*P*_(focal → dollar) = *Prob*(alternate) (Fig 2D).

The third measure builds on the second, but normalizes the rating by the probability that the event is absent when the cause is absent. This model, called Power-PC, takes the form: $TC_{PPC}\left(C\to E,u_{i}\right)=\frac{Prob(E|C)-Prob(E|\neg C)}{Prob(\neg E|\neg C)}$ [11]. It assigns a higher causal rating to the boy’s throw when the window was unlikely to shatter absent the throw. In our case, it is identical to Delta-P: *TC*_*PPC*_(focal → dollar) = *Prob*(alternate) (Fig 2D). (There is an alternate form of Power-PC which was designed specifically to assign causal attribution ratings when both the cause and outcome are known to have occurred [28]; it is the original formula divided by *Prob*(outcome ∣ cause). In both of our experiments, this alternate formula predicts a constant rating of 1 across all conditions. Hence, we do not include it in our analysis).

There are other notable models of token causal judgment that use Bayesian reasoning to infer underlying causal structures from sparse data [13, 14, 22]. These models tend to explain causal selection effects in terms of inferred asymmetries in the underlying “causal power” of variables (e.g. when some variables have higher weights in a noisy-OR structure). In our experiments, however, people already know the underlying causal structures, and the structures have no inherent causal asymmetries; the only asymmetries are in the prior probabilities of the variables. Hence, we do not include these models in our analysis. There is also an alternate class of “process” theories, which eschew counterfactuals entirely and explain token causation judgments in terms of a physical process connecting antecedent events to the outcome [6, 29–31]. These theories predict that causal judgment will not meaningfully vary with the probabilities of antecedent events in a causal system like ours; hence, we do not include them in our analysis.

### Results

People’s average ratings are shown in Fig 2E, scaled from their original range of 1 to 9 to a range of 0 to 1. Though the average ratings fell in a restricted range (about 70% were between 4-6 on the original 1-9 scale), people’s individual ratings were spread across the entire range (only 20% were between 4-6), suggesting that people were not simply choosing the midpoint of the scale out of uncertainty.

We replicated the qualitative findings from previous work. People exhibited abnormal inflation; on average, they rated drawing green (the focal variable) as more causal when drawing green was less likely. They also exhibited supersession; on average, they rated drawing green as less causal when drawing blue (the alternate variable) was less likely.

**Fig 3:**
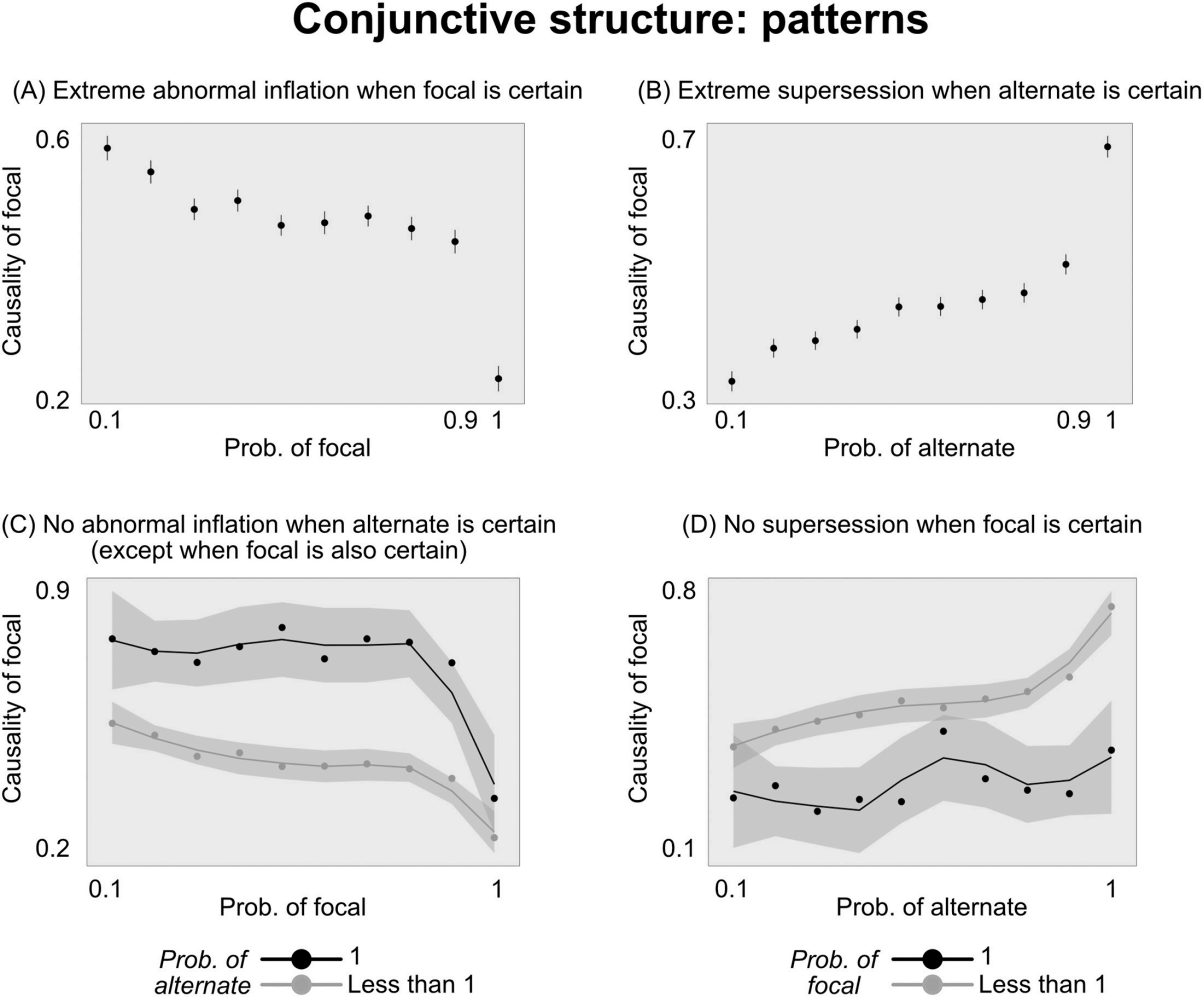
Breaking down the patterns in Experiment 1. (A) People show a larger abnormal inflation effect from *Prob*(focal) = 0.9 to *Prob*(focal) = 1. (B) People show a larger supersession effect from *Prob*(alternate) = 0.9 to *Prob*(alternate) = 1. (C) People show no abnormal inflation effect when the alternate variable is certain, except when the focal variable is also certain. (D) People show no supersession effect when the focal variable is certain (except perhaps a small effect when the alternate variable is also certain). Error bars and shaded intervals are SEM. Regression lines in (C) and (D) are estimated with Loess regression to visualize nonlinearities around certainty.

To demonstrate both of these effects statistically, we estimated a linear mixed effects model, regressing the causal ratings of green on the prior probabilities of drawing green and blue, with random intercepts and slopes for each subject. On average, people indeed rated green as more causal when its probability was lower (*b* = −0.02, *SE* = 0.002, *t* = −11.8, *p* < .001), and when the probability of drawing blue was higher (*b* = 0.03, *SE* = 0.001, *t* = 18.9, *p* < .001). (The abnormal inflation effect was notably smaller in the middle probability range, from 0.3 to 0.8 (Fig 3A). It was, however, still significant in that range (*b* = − .007, *SE* = .0024, *p* = .002)).

To rule out that these basic patterns were driven by order effects in our repeated-measures design, we analyzed how they changed from the beginning to the end of the experiment. The results did not differ much between the first and second half of trials (see S1A and S1B Fig), remained significant after controlling for trial order (*p*′*s* < 2*E* − 12), and remained significant when restricting the analysis to the first trial (*p*′*s* < 6*E* − 9), suggesting that the observed influence of *Prob*(focal) and *Prob*(alternate) on causal judgment is not due to order effects. (There was one effect of order: The abnormal inflation became slightly stronger in later trials (interaction *b* = −.0019, *SE* = 7.9*E* − 4, *t* = −2.4, *p* = .017). Future research might examine whether there are consistent effects of repeated measures on causal judgment in these scenarios).

More interesting, however, are the nonlinear patterns. We highlight four such patterns, all revolving around what happens when drawing one of the balls is certain. First, people exhibit a large jump in abnormal inflation from *Prob*(focal) = 0.9 to *Prob*(focal) = 1 (Fig 3A). In other words, when an event went from very likely to certain, this change caused an outsized drop in people’s causal rating of that event. To test this statistically, we regressed people’s causal ratings on *Prob*(focal), excluding trials where *Prob*(focal) = 1. We then used this regression model to predict people’s causal ratings for *Prob*(focal) = 1, and test whether their actual ratings when the focal variable is certain fall outside the 95% confidence interval of the regressions’s predictions. Indeed, people’s average causal rating when *Prob*(focal) = 1 is lower than what would be expected based on the ratings in the other conditions (predicted confidence interval was [0.41, 0.44]; average rating was 0.27).

Second, people exhibit a large jump in supersession from *Prob*(alternate) = 0.9 to *Prob*(alternate) = 1 (Fig 3A). In other words, when the alternate event (drawing blue) went from very likely to certain, this change caused an outsized increase in people’s causal rating of the focal event (drawing green). Using the same statistical approach as above, we find that the average rating when drawing blue is certain was higher than what would be expected based on the ratings in the other conditions (predicted confidence interval was [0.53, 0.55]; average rating was 0.70).

Third, people do not exhibit abnormal inflation when *Prob*(alternate) = 1—except for a large decrease when focal also becomes certain (dark line in Fig 3C). To test this, we regressed people’s ratings on *Prob*(focal), including only trials in which *Prob*(alternate) = 1 and excluding trials in which *Prob*(focal) = 1. Here, the probability of drawing green had no effect on people’s judgment (*p* = .77, *BF*_*null*_ = 9.0). Moreover, there was a significant interaction: People showed more abnormal inflation when *Prob*(alternate) < 1, compared to when *Prob*(alternate) = 1 (*b* = .01, *SE* = .006, *t* = 2.1, *p* = .03).

Fourth, there is an analogous effect for supersession; people do not exhibit supersession when *Prob*(focal) = 1 (except for perhaps a small effect when alternate is also certain; dark line in Fig 3D). We tested this in the same way as above, regressing people’s ratings on *Prob*(alternate), including only trials in which *Prob*(focal) = 1 and excluding trials in which *Prob*(alternate) = 1. When the focal variable was certain, the probability of the alternate variable had no effect on people’s judgment (*p* = .31, *BF*_*null*_ = 5.7). Moreover, there was a significant interaction: People showed more supersession when *Prob*(focal) < 1, compared to when *Prob*(focal) = 1 (*b* = −.01, *SE* = .006, *t* = −2.0, *p* = .04).

#### Item-level correlations between model and data

**Fig 4:**
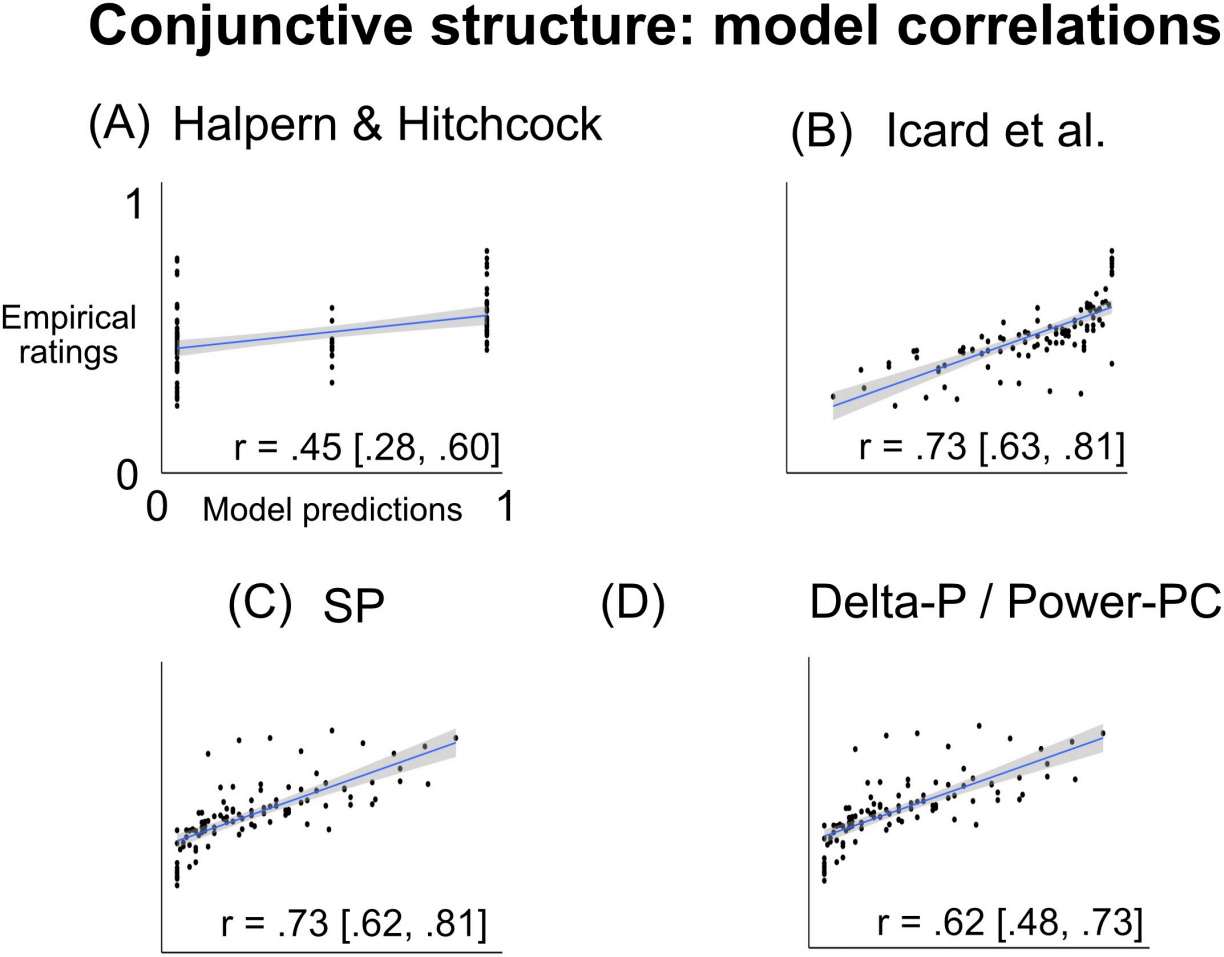
Correlation between model predictions and empirical ratings, across the various parameterizations of *Prob*(focal) and *Prob*(alternate). Each point represents one joint setting of the two probabilities (e.g. one point might capture the case where *Prob*(focal) = .7 and *Prob*(alternate) = .4); its location on the x-axis represents the model’s predicted rating for that parameterization, and its location on the y-axis represents people’s average causal ratings for that parameterization. For each model, we show a least-squares trend line (with 95% confidence intervals around it), and compute a Pearson correlation coefficient between the predicted and empirical ratings (with 95% confidence intervals in brackets). Overall, the Icard and SP models were the most accurate predictors of people’s average causal ratings across conditions.

To compare the shape of people’s response patterns to those predicted by the models, we computed the correlation between the empirical ratings and each model’s predictions, across settings of *Prob*(focal) and *Prob*(alternate) (Fig 4). Concretely, we computed (a) each model’s predicted causal rating and (b) people’s average causal rating, for each point in the joint parameterization of *Prob*(focal) and *Prob*(alternate). For example, when *Prob*(focal) = .7 and *Prob*(alternate) = .4, the Icard model predicts a causal rating of 0.58, and people’s average causal rating was .47. Since there were 10 possible settings of *Prob*(focal) and 10 possible settings of *Prob*(alternate), this gives us 100 pairs of data points (the predicted rating and people’s actual average rating) for each model. We then estimated a Pearson correlation coefficient between the predicted and empirical ratings across these 100 conditions, giving us an estimate of how well each model predicted variation in people’s average ratings across conditions. Icard et al.’s model and SP showed the highest correlation with people’s ratings (*r*′*s* = .73), and did not differ significantly from each other (*p* = .9). Icard et al.’s model was significantly more correlated with people’s ratings than the Halpern & Hitchcock model or the Delta-P/Power-PC model (Williams’s test, *p*′*s* < .05). SP was significantly more correlated with people’s ratings than the Halpern & Hitchcock model (*p* < .05), and marginally more correlated than the Delta-P/Power-PC model (*p* = .08).

The Icard model and SP both scored well by predicting the linear trends of abnormal inflation and supersession. However, neither correctly characterized the overall nonlinear patterns in people’s ratings. In particular, none captured the effects of certainty described above.

### Discussion

We characterized people’s quantitative causal ratings across the free parameters of a conjunctive causal system. As expected, people exhibit both abnormal inflation, where their causal rating of the focal variable (green) increased as it became rarer, and supersession, where their causal rating of the focal variable decreased as the alternate (blue) became rarer.

Moreover, people showed clear nonlinear patterns in judgment when the candidate causes were certain to occur. Specifically, they showed an unexpectedly strong drop in their causal rating of the focal variable when it was certain to occur, and an unexpectedly strong increase when the alternate was certain to occur. Moreover, the effect of abnormal inflation disappeared when the alternate was certain to occur, and the effect of supersession disappeared when the focal variable was certain to occur. These patterns are not well-described by extant models.

## Experiment 2: Disjunctive scenario

**Fig 5:**
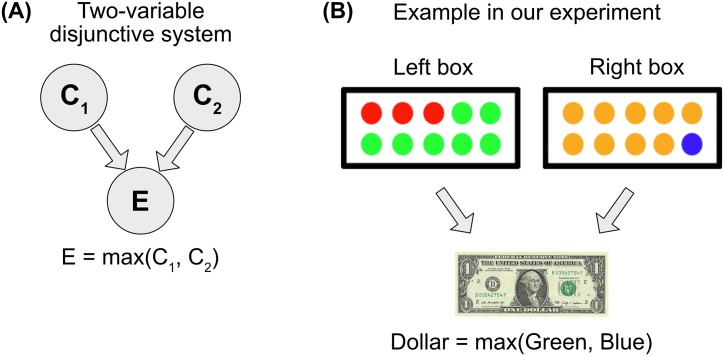
Causal structure (A) and vignette (B) used in Experiment 2.

Experiment 2 was identical to Experiment 1, except the causal structure was disjunctive instead of conjunctive (Fig 5A). We used the same vignette, but told participants that Joe won a dollar if either a green ball was drawn from the left box, *or* a blue ball was drawn from the right box (Fig 5B).

On each trial, participants were told that Joe drew both a green and blue ball, and won the dollar. As before, we parametrically manipulated the prior probabilities of drawing green (*Prob*(focal = 1)) and blue (*Prob*(alternate = 1)) from 0.1 to 1, and asked people the extent to which they agreed that Joe’s drawing the green ball caused him to win the dollar. (As in the Experiment 1, we did not allow the probabilities of focal or alternate to be zero. This kept Experiment 2 closer in form to Experiment 1. However, in the disjunctive structure of Experiment 2, it would have been possible to include zero-probability cases; future work should examine people’s causal judgment in these cases).

### Methods

All participants were recruited through Amazon Mechanical Turk, and gave informed consent. We ran *N* = 1013 subjects, each of which gave 5 ratings (for a total of 5039 ratings and an average of about 50 ratings per probability setting). The experiment again took about 3 minutes to complete, and participants were paid $0.35 each.

### Theoretical predictions

In the disjunctive structure, the qualitative predictions from past research were (a) that participants would show abnormal *deflation* (making an event rarer would make it seem less causal), and (b) that participants would not show supersession (making the alternate event rarer would not affect causal judgment; see [3, 21]). For more precise, quantitative predictions, we turned to the same models as in Experiment 1.

#### Halpern & Hitchcock

Recall that in Halpern & Hitchcock’s model [4], a variable is considered a cause if the world we must imagine to judge whether it was necessary—its “witness” world—is more normal than the actual world. (As before, we assume that, in our task, normality ordering is determined by the probabilities of drawing green and blue). In Experiment 2, to judge whether drawing a green ball was necessary for winning the dollar, we must consider a possible world—call it *w*_focal_—in which neither a green nor a blue ball was drawn, because it is only when alternate = 0 that focal could have been necessary. (For more details of how their model treats overdetermined cases like Experiment 2, see [4]). Hence, the model predicts that the causal status of focal will depend on whether that world—*w*_focal_—is more or less normal than the actual world.

To determine the precise predictions, there are three cases to consider. First, consider the case where both *Prob*(focal) and *Prob*(alternate) are less than 0.5. In this case, *w*_focal_ (where neither ball is drawn) is surely more normal than the actual world (where both balls are drawn), and the model predicts that focal will be judged causal. Moreover, as long as *Prob*(focal) < .5 and *Prob*(alternate) < .5, the extent to which focal is judged a cause is not predicted to vary with either probability; the only other candidate cause, alternate, has exactly the same witness world, and so the relative causality of focal will not vary as the normality of that witness world changes. Hence, in this case, the model assigns focal a constant causal rating, which we label *x*_*high*_.

Similarly, if *Prob*(focal) and *Prob*(alternate) are both greater than 0.5, then *w*_focal_ is less normal than the actual world, and the model predicts that focal will be judged noncausal. Hence, in this case, the model assigns focal a causal rating of zero.

The third and hardest case is when one of the two variables is likely, and the other is rare—that is, when *Prob*(focal) < .5 and *Prob*(alternate) > .5, or when *Prob*(focal) > .5 and *Prob*(alternate) < .5. Here, there are at least two ways of instantiating Halpern & Hitchcock’s model. From one perspective, the actual world and *w*_focal_ are, in this case, incomparable; one variable takes on its more typical value, but another takes on its less typical value, and the model offers no machinery for integrating those two facts. This is the view adopted in the original paper [4]. When the actual world cannot be compared to the witness world, the model assigns a causal rating of zero—and hence focal will be judged noncausal.

But it is plausible that, in our experiment, people would rank the normality of a world according to the *total* probability of its variables. On this view, focal will only be considered causal if the total probability of the actual world (where both balls are drawn)— *Prob*(focal) ⋅ *Prob*(alternate)—is less than the total probability of the witness world (where neither ball is drawn)— *Prob*(¬focal) ⋅ *Prob*(¬alternate). This condition reduces to *Prob*(focal) + *Prob*(alternate) < 1.

This leaves us with two potential response profiles, HH1 and HH2:

$$\begin{array}{c} TC_{HH1}\left(\text{focal}\to \text{dollar}\right)=\{\begin{array}{l} x_{high}\;if\;Prob\left(\text{focal}\right),\;Prob\left(\text{alternate}\right)<.5\\ 0\;otherwise \end{array} \end{array}$$

$$\begin{array}{c} TC_{HH2}\left(\text{focal}\to \text{dollar}\right)=\{\begin{array}{l} x_{high}\;if\;Prob\left(\text{focal}\right),\;Prob\left(\text{alternate}\right)<1\\ 0\;otherwise \end{array} \end{array}$$

**Fig 6:**
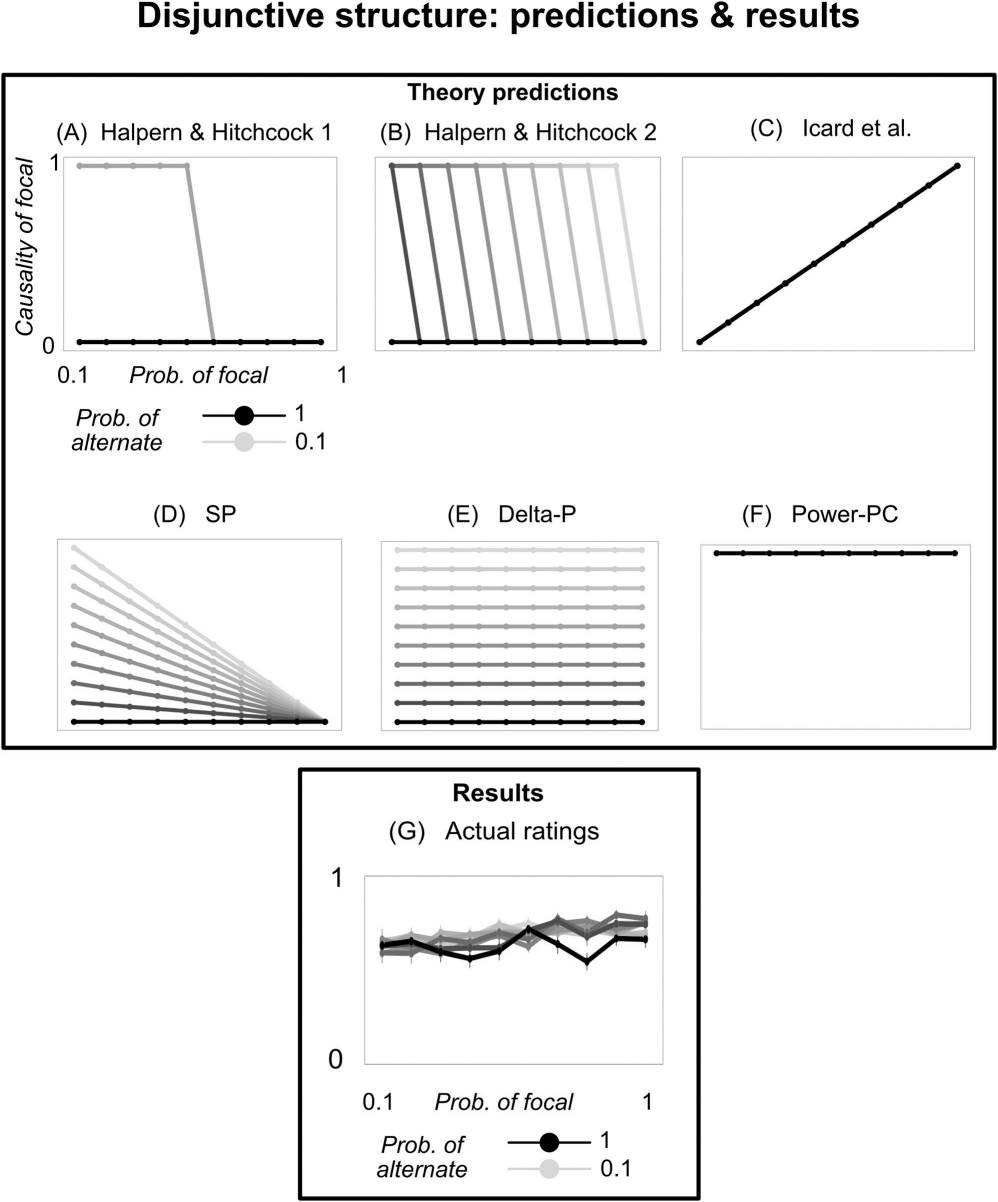
Predictions and results from Experiment 2. (A-F) Predictions from the models we analyze. The x-axis indicates the probability of drawing a green ball; line color indicates the probability of drawing a blue ball; and the y-axis indicates focal’s predicted causal rating. (G) Empirical results; y-axis indicates people’s agreement that drawing a green ball caused the outcome. Error bars are SEM.

Below, we consider both variations of the model. Their predictions are depicted in Fig 6A and 6B, assuming *x*_*high*_ = 1.

#### Icard et al.

Recall that, according to the model of [3]:

$$\begin{array}{l} TC_{Icard}\left(\text{focal}\to \text{dollar}\right)=\\ \;Prob\left(\neg \text{focal}\right)\cdot \;Prob\left(\text{focal}\;\text{was}\;\text{necessary}\right)+\\ \;Prob\left(\text{focal}\right)\cdot Prob\left(\text{focal}\;\text{would}\;\text{be}\;\text{sufficient}\right) \end{array}$$

In the structure of Experiment 2, focal is not necessary in the actual world, and would always be sufficient. Hence, this equation reduces to:

$$\begin{array}{l} TC_{Icard}\left(\text{focal}\to \text{dollar}\right)=Prob\left(\neg \text{focal}\right)\cdot 0+Prob\left(\text{focal}\right)\cdot 1\\ \;=Prob\left(\text{focal}\right) \end{array}$$

The Icard model’s predictions are shown in Fig 6C.

#### Classic causal strength measures

In the disjunctive structure of Experiment 2, the three causal strength measures considered above reduce to (Fig 6D–6F):

$$\begin{array}{l} TC_{SP}\left(\text{focal}\to dollar\right)=Prob\left(\neg \text{focal}\right)\cdot Prob\left(\neg \text{alternate}\right)\\ TC_{DP}\left(\text{focal}\to dollar\right)=Prob\left(\neg \text{alternate}\right)\\ TC_{Power-PC}\left(\text{focal}\to \text{dollar}\right)=1 \end{array}$$

### Results

People’s average ratings are shown in Fig 6G. As predicted by previous qualitative work [3], people exhibited abnormal deflation; they rated focal as less causal when it was rarer (*b* = .01, *SE* = .002, *t* = 8.8, *p* < .00001). However, contrary to previous work [3, 21], there was also a “reverse” supersession effect: People rated focal as less causal when alternate was more common (*b* = −.005, *SE* = .001, *t* = −3.7, *p* = .0003). (As in Experiment 1, these patterns were not a spurious result of repeated measures; they were similar in both the first and second half of trials (see S1C and S1D Fig), remained significant after controlling for trial order (*p*′*s* < .005), and remained significant when restricting analysis to the first trial (*p*′*s* < .02)).

**Fig 7:**
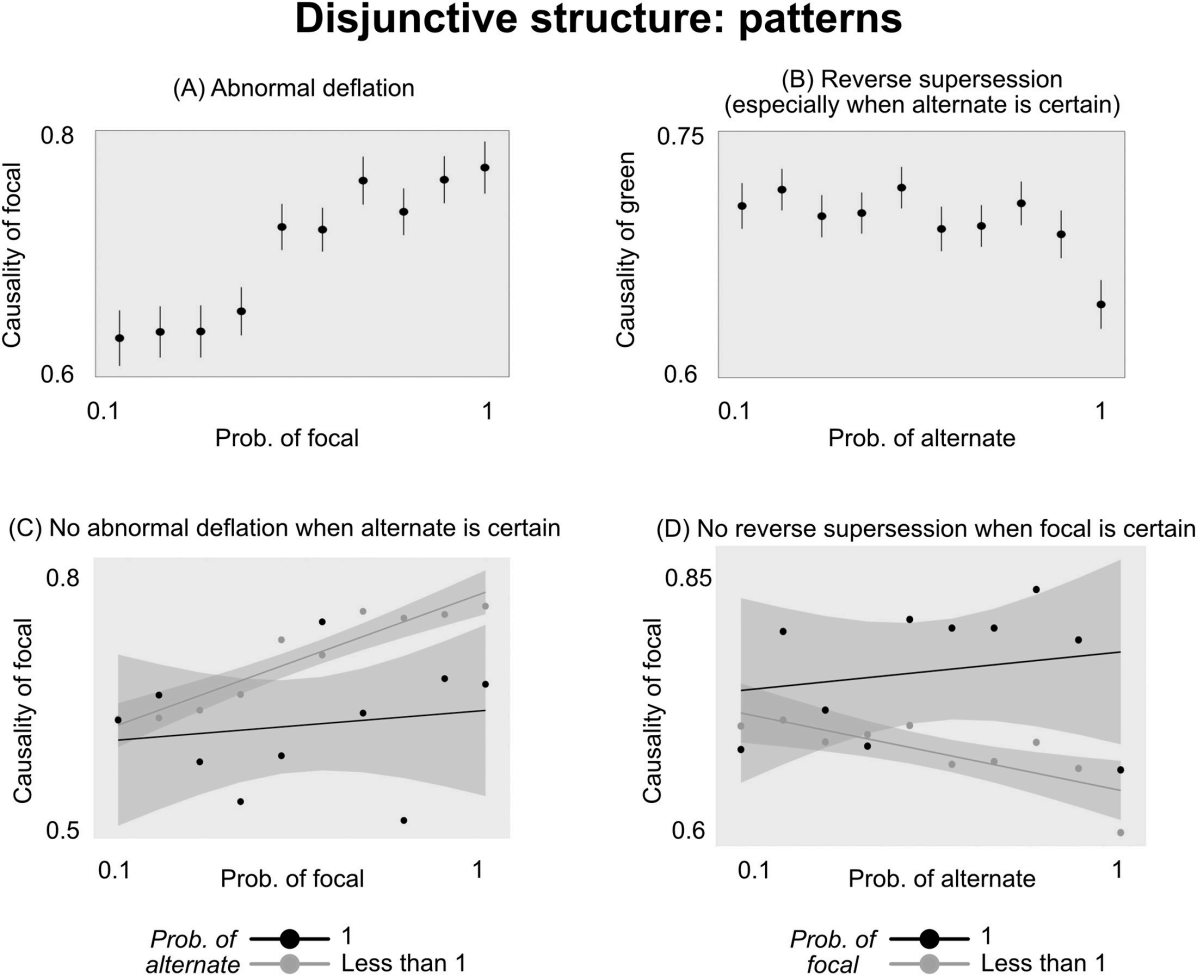
Breaking down the patterns in Experiment 2. (A) People exhibit a linear effect of abnormal deflation. (B) People show a larger reverse supersession effect from *Prob*(alternate) = 0.9 to *Prob*(alternate) = 1, although the effect is still significant after excluding the trials where drawing blue is certain. (C) People don’t show abnormal deflation when drawing blue is certain. (D) People don’t show reverse supersession when drawing green is certain. Error bars and shaded intervals are SEM; regression lines in (C) and (D) are estimated with standard sum-of-squares.

We dissect these effects further in Fig 7. The abnormal deflation effect is largely linear; unlike with abnormal *inflation* in conjunctive structures, here there is no large jump from *Prob*(focal) = 0.9 to *Prob*(focal) = 1 (Fig 7A). The reverse supersession effect, however, does show a nonlinearity: It is strongest when drawing blue becomes certain (Fig 7B). We tested this statistically in the same way as in Experiment 1, and found that people’s ratings when *Prob*(alternate) = 1 were significantly lower than expected based on a linear model of the other conditions (predicted confidence interval was [.67, .70]; average rating was .64). Noteably, the reverse supersession effect, though small, was still significant after excluding the condition when drawing blue was certain (*b* = −.004, *SE* = .002, *t* = −2.4, *p* = .02).

There were two other nonlinear patterns that mirrored the patterns in Experiment 1. Recall that, in Experiment 1, people stopped exhibiting abnormal inflation when drawing blue was certain, and stopped exhibiting supersession when drawing green was certain (with certain exceptions discussed above). In Experiment 2, we find an analogous pattern: People stop exhibiting abnormal deflation when drawing blue is certain (Fig 7C), and stop exhibiting reverse supersession when drawing green is certain (Fig 7D). (The pattern of causal judgment across conditions appears unstable when *Prob*(focal) or *Prob*(alternate) = 1 (black points in Fig 7C and 7D). This instability is likely due to the small number of subjects in those conditions; each point along the black line represents an average of 50 ratings, and the standard errors are large enough to be consistent with a linear pattern. Of course, we cannot be sure; future research should use larger samples in those conditions).

These visual observations were confirmed statistically. When *Prob*(alternate) = 1, there was no effect of *Prob*(focal) on people’s ratings (*p* = .75, *BF*_*null*_ = 9.5); moreover, the effect of *Prob*(focal) was significantly stronger when *Prob*(alternate) < 1 compared to when *Prob*(alternate) = 1 (interaction *b* = −.01, *SE* = .005, *t* = −2.3, *p* = .02). Similarly, when *Prob*(focal) = 1, there was no effect of *Prob*(alternate) on people’s ratings (*p* = .55, *BF*_*null*_ = 8.5); moreover, the effect of *Prob*(alternate) was stronger when *Prob*(focal) < 1 compared to when *Prob*(focal) = 1, although in this case the interaction was marginal (*b* = .01, *SE* = .005, *t* = 1.7, *p* = .095).

#### Model correlations

**Fig 8:**
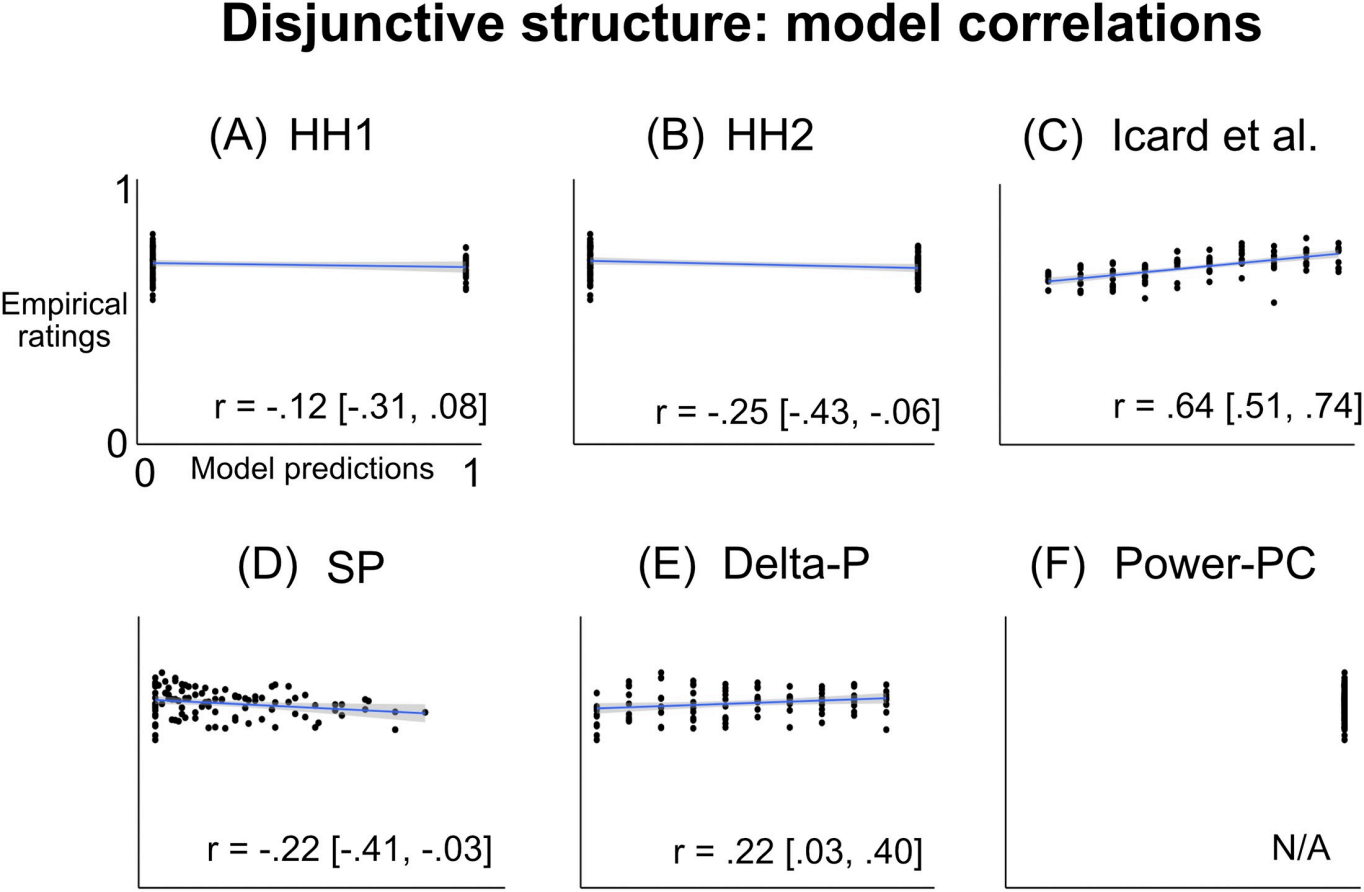
Correlation between model predictions and empirical ratings for Experiment 2. Brackets show 95% confidence intervals for Pearson correlation coefficients. The correlation for Power-PC cannot be calculated because it predicts a constant response profile.

The correlations between the model predictions and people’s ratings across conditions are shown in Fig 8. The Icard model showed by far the best correlation with people’s ratings (*r* = .64, *p* < .00001); It was significantly more correlated with the empirical ratings than the next-best model (*p* = .0005). As in Experiment 1, however, it did not capture all the major patterns in people’s responses—most notably, it did not capture the reverse supersession effect in Fig 7B, or either of the nonlinear patterns in Fig 7C and 7D.

### Discussion

We characterized people’s quantitative causal ratings across the free parameters of a disjunctive causal system. As predicted by the Icard model [3], people exhibited abnormal deflation, where their causal rating of the focal variable decreased as it became rarer. Surprisingly, they also exhibited a “reverse” supersession effect, where their causal rating of the focal variable increased as the alternate variable became rarer.

Moreover, people’s nonlinear patterns mirrored those in Experiment 1: They stopped exhibiting abnormal deflation when the alternate variable was certain to occur, and stopped exhibiting reverse supersession when the focal variable was certain to occur. These nonlinearities are not well-described by extant models.

## General discussion

We presented people with two basic causal systems, and elicited people’s causal judgments across parametric manipulations of two key parameters in those systems: the prior probability of the focal event (whose causal status was being rated), and the the prior probability of an alternate event (that was either necessary or sufficient for the outcome). These experiments replicated three previously established qualitative effects—abnormal inflation and supersession in the conjunctive structure, abnormal deflation in the disjunctive structure—and revealed a novel effect of reverse supersession in the disjunctive structure. Moreover, people exhibited a constellation of nonlinear patterns that were strikingly consistent across the two experiments. We discuss these results in turn (see Table 1 for a summary).

**Table 1:** Summary of the empirical findings from the two experiments.

Table of effects	*In conjunctive structure…*	*In disjunctive structure…*
*As focal becomes more likely:*	People rate focal as less causal (abnormal inflation)	People rate focal as more causal (abnormal deflation)
*As alternate becomes more likely:*	People rate focal as less causal (supersession)	People rate focal as more causal (reverse supersession)
*As focal becomes certain:*	People show stronger abnormal inflation, and stop showing supersession.	People stop showing reverse supersession.
*As alternate becomes certain:*	People show stronger supersession, and stop showing abnormal inflation.	People show stronger reverse supersession, and stop showing abnormal deflation.

### Reverse supersession

The original supersession effect is that, in conjunctive structures, a focal event is rated less causal when an alternate event becomes rarer; the alternate event “supersedes” the focal event [21]. This effect was only supposed to apply, however, to conjunctive structures; two previous papers predicted and found that there would be no supersession in disjunctive structures [3, 21].

In contrast to those papers, we do find a kind of supersession effect in the disjunctive structure of Experiment 2. The effect is reversed: The focal event is rated more causal when the alternate event becomes rarer (or, equivalently, the focal event is rated less causal when the alternate event becomes more common). Given the logic of supersession, this reversal is unsurprising. In disjunctive structures, events become causally preferred when they are more common (the “abnormal deflation” effect). It makes sense, then, that if the alternate event were to supersede the focal event, it would do so when it was common, not rare.

What *is* surprising is that, in contrast to previous work, the alternate event supersedes the focal event at all in the disjunctive structure. This result should be interpreted with caution. The effect is mostly driven by the condition where the alternate event is certain to occur (when excluding this condition, the effect is tiny, though still significant). Nonetheless, it is useful to consider how this result might be reconciled with the null findings in previous papers [3, 21]. We see three possibilities. First, in our data, the supersession effect in disjunctive structures was indeed much weaker than the corresponding supersession effect in conjunctive structures. This is in line with the findings from previous work. Second, previous papers relied on qualitative manipulations of the probability of the alternate event; this may have made it difficult to detect the subtle effect reported here. Third, previous papers used different types of vignettes; it is possible that supersession in disjunctive structures is unique to the type of experimental manipulation used here. Future work should test for disjunctive supersession in a wider range of vignettes.

### Nonlinear patterns around certainty

Across most of the parameter space, the effects of prior probabilities on causal judgment appear largely linear and additive. That is, the prior probabilities of the focal and alternate events each have a mostly-linear influence on causal judgment, and do not interact much. The main exception is when the probability of either event approaches 1. When one of the events is certain to occur, people exhibit a constellation of departures from the linear, additive baseline.

First, three of the four primary effects—abnormal inflation and supersession in Experiment 1, reverse supersession in Experiment 2—become very strong when the relevant events become certain. For instance, in Experiment 1, although the focal variable (drawing a green ball) is considered less and less causal as it becomes more common, the drop in causal rating from *Prob*(focal) = 0.9 to *Prob*(focal) = 1 is much larger than the drops between other conditions (See Fig 3A).

Discontinuities in causal judgment when an event is certain have been discussed before. Perhaps most prominently, Cheng & Novick [2] argued that, when an event is certain to occur, people treat it as an “enabling condition”, not a cause (see also [6, 30, 32–34]). The categorical distinction between cause and enabling condition may help explain why, in the conjunctive structure, people are particularly hesitant to label the focal event a cause when it was certain to occur. However, these “enabling condition” theories have difficulty explaining why people judged the certain events *somewhat* causal (instead of at zero causality), and why people judged the certain events to be highly causal in the disjunctive structure.

Another possibility is that these nonlinearities result from a domain-general feature of people’s probabilistic reasoning: People, in general, treat the jump from almost-certain to certain as more important than other equivalently sized jumps in probability [35, 36]. This finding, known as the “certainty effect”, could help explain why the patterns in causal judgment become more pronounced when an event becomes certain. (Similarly, the outsized effect of certainty might be explained by people applying certain transformations, such as a logit transformation, to their representation of the probabilities).

Both of these perspectives, however, have trouble accounting for the second set of departures from the linear, additive baseline: People stop exhibiting all the effects described here (abnormal inflation/deflation, supersession) when the *other* event is certain to occur. Concretely, the prior probability of the focal event stops influencing people’s causal judgment when the alternate event is certain to occur, and the probability of the alternate event stops influencing people’s judgment when the focal event is certain to occur. These results, which are consistent across both experiments should be further examined in future research.

The nonlinearities observed in our experiments are primarily about what happens when one of the events becomes certain to occur. It is also possible that there are similar nonlinearities when one of the events becomes certain *not* to occur, or when the probability gets close to zero. This is a ripe question for future research.

### Comparing extant models

Out of the models analyzed here, the response profile predicted by Icard et al. was consistently the most similar to people’s ratings [3]. This is perhaps not surprising, since Icard et al. built their model with some of our primary effects—i.e. abnormal selection and supersession—in mind. Still, it is satisfying that their model, which was designed principally to capture qualitative effects, roughly tracks the overall shape of people’s quantitative responses in our experiments. However, no model analyzed here captured all the nonlinear patterns in people’s judgments, highlighting the need for further theorizing.

### Conclusion

People’s causal judgments are influenced by the prior probabilities of the candidate causes. Here, we find that people show systematic patterns of judgment across parametric manipulations of those probabilities, in a way that is not fully characterized by existing theories. We hope this spurs further research into the quantitative nature of token causal judgment.

## Supporting information

S1 Fig The influence of *Prob*(focal) and *Prob*(alternate) on causal judgment, in the first and second half of trials (each person made judgments in five scenarios; “first half” is scenarios 1-2, “second half” is scenarios 3-5). (A-B) The effects in Experiment 1; (C-D) the effects in Experiment 2. People show roughly similar patterns across the two halves, suggesting that the influence of *Prob*(focal) and *Prob*(alternate) is not primarily due to order effects. (The only significant order effect was for abnormal inflation in (A), which got slightly stronger in the second half (interaction *b* = −.0019, *SE* = 7.9*E* − 4, *t* = −2.4, *p* = .017).)
(TIFF)
