# Supplementary figures and images for: Quantitative causal selection patterns in token causation
Source: PLoS One. 2019 Aug 1;14(8):e0219704. doi: 10.1371/journal.pone.0219704 (PMC6675094; doi:10.1371/journal.pone.0219704)

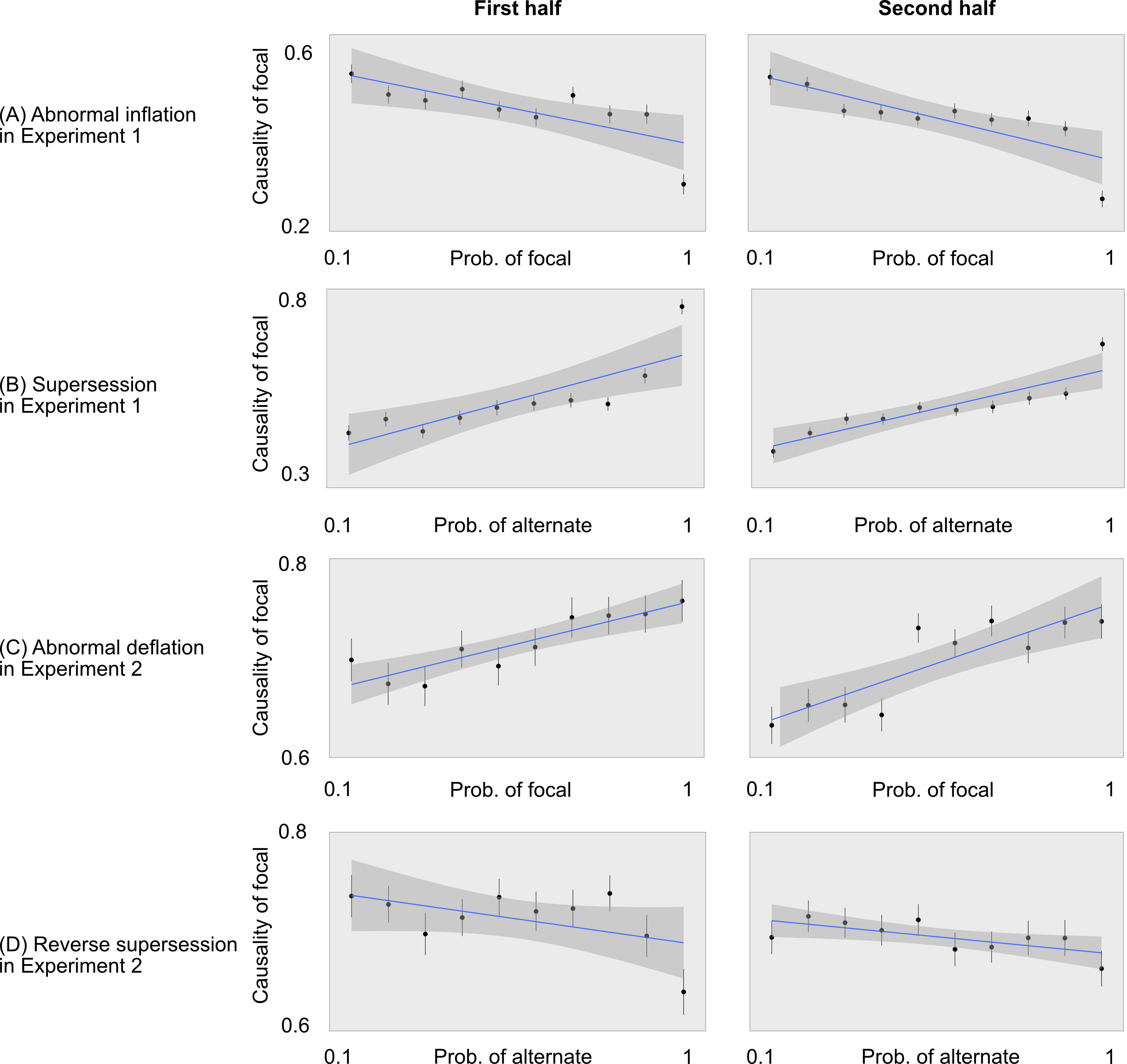

Supplement: S1 Fig — (A-B) The effects in Experiment 1; (C-D) the effects in Experiment 2. People show roughly similar patterns across the two halves, suggesting that the influence of Prob(focal) and Prob(alternate) is not primarily due to order effects. (The only significant order effect was for abnormal inflation in (A), which got slightly stronger in the second half (interaction b = −.0019, SE = 7.9E − 4, t = −2.4, p = .017).) (TIFF) [file pone.0219704.s001.tiff]
